# Automated freeze-thaw cycles for decellularization of tendon tissue - a pilot study

**DOI:** 10.1186/s12896-017-0329-6

**Published:** 2017-02-14

**Authors:** Susanne Pauline Roth, Sina Marie Glauche, Amelie Plenge, Ina Erbe, Sandra Heller, Janina Burk

**Affiliations:** 1 Large Animal Clinic for Surgery, University of Leipzig, An den Tierkliniken 21, Leipzig, 04103 Germany; 2 Saxonian Incubator for Clinical Translation, University of Leipzig, Philipp-Rosenthal-Strasse 55, Leipzig, 04103 Germany; 3 Tierklinik Kaufungen, Pfingstweide 2, Kaufungen, 34260 Germany; 4 Department of Pathology and Laboratory Medicine, Tulane University, New Orleans, USA; 5 Institute of Veterinary Physiology, University of Leipzig, An den Tierkliniken 7, Leipzig, 04103 Germany

**Keywords:** Regenerative medicine, Tissue engineering, Tendon, Horse, Automation, Decellularization, Controlled rate freezer

## Abstract

**Background:**

Decellularization of tendon tissue plays a pivotal role in current tissue engineering approaches for in vitro research as well as for translation of graft-based tendon restoration into clinics. Automation of essential decellularization steps like freeze-thawing is crucial for the development of more standardized decellularization protocols and commercial graft production under good manufacturing practice (GMP) conditions in the future.

**Methods:**

In this study, a liquid nitrogen-based controlled rate freezer was utilized for automation of repeated freeze-thawing for decellularization of equine superficial digital flexor tendons. Additional tendon specimens underwent manually performed freeze-thaw cycles based on an established procedure. Tendon decellularization was completed by using non-ionic detergent treatment (Triton X-100). Effectiveness of decellularization was assessed by residual nuclei count and calculation of DNA content. Cytocompatibility was evaluated by culturing allogeneic adipose tissue-derived mesenchymal stromal cells on the tendon scaffolds.

**Results:**

There were no significant differences in decellularization effectiveness between samples decellularized by the automated freeze-thaw procedure and samples that underwent manual freeze-thaw cycles. Further, we inferred no significant differences in the effectiveness of decellularization between two different cooling and heating rates applied in the automated freeze-thaw process. Both the automated protocols and the manually performed protocol resulted in roughly 2% residual nuclei and 13% residual DNA content. Successful cell culture was achieved with samples decellularized by automated freeze-thawing as well as with tendon samples decellularized by manually performed freeze-thaw cycles.

**Conclusions:**

Automated freeze-thaw cycles performed by using a liquid nitrogen-based controlled rate freezer were as effective as previously described manual freeze-thaw procedures for decellularization of equine superficial digital flexor tendons. The automation of this key procedure in decellularization of large tendon samples is an important step towards the processing of large sample quantities under standardized conditions. Furthermore, with a view to the production of commercially available tendon graft-based materials for application in human and veterinary medicine, the automation of key procedural steps is highly required to develop manufacturing processes under GMP conditions.

## Background

Decellularization of natural tissues offers promising opportunities as a multi-purpose tool in the field of tissue engineering [1]. Particularly, the challenging creation of decellularized biological scaffolds with a preserved tissue-specific extracellular matrix (ECM) composition provides a crucial field of application. The importance of biological scaffolds with a naturally structured ECM is based on their similarity to in vivo conditions for cell attachment, proliferation, and differentiation while maintaining biomechanical functionality and biocompatibility.

Among biological scaffolds used for clinical application in regenerative medicine and for current tissue engineering approaches, decellularized tendon tissue plays a pivotal role. This is due to a high and still increasing incidence of tendon pathologies, such as injuries of the Achilles tendon and traumatic rupture of the anterior cruciate ligament, in an aging population with growing sporting ambitions [2–4]. Moreover, currently available treatment options for tendinopathies are often not evidence-based and do not effectively prevent re-injury, placing a heavy burden on the health care system and the social economy [5]. Therefore, tissue engineering involving decellularization techniques aims not only to prepare 3D-culture models for in vitro research [6], but also to produce clinically available tissue grafts for the reconstruction of musculoskeletal defects [1, 7] with the ultimate goal to translate tendon restoration into clinics.

The distinguishing and so far inimitable feature of site-specific homologous tissue for graft-based tendon reconstruction is its highly specific ECM composition, reflecting original biochemical and biomechanical tissue characteristics at its best. Since synthetic biomaterials turned out to be more or less inadequate for tendon reconstruction, biological tendon grafts including auto-, allo- and xenografts are considered as gold standard for tendon repair [1, 5]. Especially due to donor site morbidity, abundantly available allo- and xenografts are favoured for the development of scaffolds reflecting natural tendon ECM composition [1, 8]. In order to avoid rejection after implantation due to cell associated immune response, and to allow re-seeding procedures, decellularization is an essential step towards the application of appropriate tendon-derived scaffolds [8].

To date, different protocols for decellularization of tendon tissue of various species have been evaluated, using physical as well as chemical and/or enzyme-based methods. However, published protocols for decellularization of natural tendon tissue provide little insight regarding the impact of specific decellularization steps [9]. Physical treatments, like agitation or sonication, mechanical massage or pressure, or freeze-thaw cycles, are the most commonly used techniques to disrupt cell membranes, detach cells within their ECM network and allow further rinsing to remove cell remnants [5, 10]. Whereas the sole application of freeze-thawing was reported as insufficient for tendon decellularization [10], Burk et al. reported a significantly more effective decellularization of large tendon samples by combining repetitive freeze-thaw cycles with a detergent treatment when compared to a sole application of detergents [11]. Although procedural steps such as freeze-thaw cycles are considered as key procedure, there is no consensus on a certain protocol or standardized parameters for evaluation of their effectiveness [12–14].

Considering safety of application in clinical use and the necessity of commercial graft production under good manufacturing practice (GMP) conditions, standardization and accurate documentation of all procedural steps are crucial. Furthermore, identical processing of large sample quantities will be required, which can only be achieved by automating key procedures of decellularization. Therefore, our study aimed to directly compare the influence of manual and automated freeze-thaw cycles on the decellularization effectiveness in equine superficial digital flexor tendons, based on a previously described decellularization protocol [11]. Further, we intended to evaluate the impact of two different cooling as well as heating rates in automated freeze-thaw cycles with regard to decellularization effectiveness. To our knowledge, this is the first description of using automated freeze-thaw cycles for tissue decellularization, leading to standardization and optimization of decellularization protocols.

## Methods

### Study design

Freshly collected equine superficial digital flexor tendon samples (*n* = 10) were used to assess the effectiveness of automated freeze-thawing as part of an otherwise established decellularization protocol. Two different freeze-thawing protocols were carried out using a controlled rate freezer (PLANER® Kryo 360 - 1.7). Additionally, a manual freeze-thawing procedure in accordance with an established protocol was done.

In all three protocols (Auto-Protocol 1 and 2 and Manual-Protocol, respectively), after decellularization was initiated by repetitive freeze-thaw cycles, the same subsequent procedure of detergent treatment was applied. Parameters to evaluate the effectiveness of decellularization included histologically visible nuclei and DNA content. In addition, further tendon samples (*n* = 3) were re-seeded with allogeneic equine adipose tissue-derived mesenchymal stromal cells (AT-MSC) to evaluate cytocompatibility.

### Sample collection

Fresh cadaver limbs from middle-sized warmblood horses obtained at a local abattoir served as source of tendon samples. The aseptically performed collection of tendon specimens from the palmar/plantar aspect of the mid-metacarpus/-metatarsus was done within 6 h after slaughter under sterile working conditions. During this period of time, the skin of the cadaver limbs remained closed to keep the tendon tissue sterile. To further prevent bacterial contamination, the recovered tendon samples were placed in phosphate buffered saline (PBS; Sigmal Aldrich) supplemented with 2% penicillin-streptomycin (Sigma Aldrich) and 0.1% gentamycin (Carl Roth) for overnight storage at 4 °C. Immediately before automated or manual freeze-thawing, the stored tendon specimens were washed 5 min each in PBS and 70% ethanol two times.

### Decellularization

From each 8 cm long tendon sample, 2 cm were separated to serve as internal control, whereas the remaining 6 cm were divided into three equal 2 cm long parts. The cross-sectional dimension of the tendons were left unchanged, measuring roughly 1.5 cm × 0.5 cm. Each of the latter tendon parts underwent decellularization according to one of three different protocols (Table 1). In group 1 and 2 (Auto-Protocol 1 and 2), automated freeze-thaw cycles with controlled cooling and heating rates differing in the applied temperature change per minute were performed. Safe and sterile placement of tendon samples inside the controlled rate freezer was ensured by using plastic sampling bags (Carl Roth; *n* = 7 tendon samples) or 15 ml conical centrifuge tubes (VWR; *n* = 3 tendon samples). For this purpose, the tube holders of the controlled rate freezer had been modified in collaboration with the manufacturer. Group 3 samples (Manual-Protocol) were subjected to manual freeze-thaw cycles including five cycles of 2 min freezing in liquid nitrogen and 10 min thawing in PBS at 37 °C. Further decellularization was carried out at room temperature as well as under continuous agitation and was performed in the same way for all three groups. For the purpose of rinsing and induction of cell lysis by osmotic effects, all samples were incubated in hypotonic solution (distilled water) for 48 h. Afterwards, tendon samples were incubated for 48 h in Tris buffer (Carl Roth) (pH 7.6) containing 1% Triton X-100 (Carl Roth). Decellularization was completed by the following washing steps: 2 × 15 min in distilled water, 24 h in cell culture medium [DMEM 1 g glucose/L (Thermo Fisher Scientific) supplemented with 10% fetal bovine serum (FBS; Sigma Aldrich), 1% penicillin-streptomycin (Sigma Aldrich), 0.1% gentamycin (Carl Roth)], and again 24 h in PBS. The performed incubation in cell culture medium served not only the purpose of rinsing to remove cellular remnants and residual chemicals, but also to prepare the scaffolds for cell culture optimally.

**Table 1:** Sample groups and decellularization protocols

Group	Protocol	Decellularization procedures				
		5 repetitions of freeze-thawing				Further treatment
		Cooling	Freeze hold	Heating	Thaw hold	
1	Auto-Protocol 1	-50 °C per min	3 min at -80 °C	+50 °C per min	10 min at +20 °C	48 h distilled water 48 h 1% Triton X-100 Washing steps
2	Auto-Protocol 2	-20 °C per min	3 min at -80 °C	+20 °C per min	10 min at +20 °C	
3	Manual-Protocol	Manual transfer	2 min in liquid nitrogen	Manual transfer	10 min in 37 °C PBS	
	Control	No treatment				

Equine superficial digital flexor tendon samples of group 1 and group 2 were processed by automated freeze-thaw cycles, differing in the performed cooling and heating rates (Auto-Protocol 1 and Auto-Protocol 2). Both of the applied cooling and heating rates describe a temperature change per unit time. For Auto-Protocol 1 as well as for Auto-Protocol 2 the maximum reached temperature was + 20 °C (thaw hold for 10 min) and the minimum reached temperature was -80 °C (freeze hold for 3 min). All temperature regulations of the automated freeze-thaw cycles were carried out by a controlled rate freezer (PLANER® Kryo 360–1.7) that utilizes liquid nitrogen to adjust temperature. Group 3 included manual freeze-thaw cycles. Further steps of decellularization were the same for all sample groups. Tendon samples classified as internal control underwent no decellularization

### Assessment of decellularization effectiveness

#### Histology and nuclei count

For this analysis, one piece of tendon tissue was obtained from the centre of each sample. These pieces were fixed in 4% paraformaldehyde and embedded in paraffin. Hematoxylin and eosin staining of two longitudinal 6 μm sections per sample followed. Three randomly chosen regions of the prepared slides were photographed at 20 × magnification (Leica DMi1, Leica MC 170HD, LAS V4.5 Software, Leica Microscope CMS GmbH) and visible cell nuclei were counted. Results from visible nuclei count were normalized to the respective internal controls and are given as percentages relative to controls.

#### DNA content

To calculate DNA content, pieces of tendon tissue recovered from the centre of each sample were subjected to papain digestion. For this purpose, 200 mg of each sample were minced into 1 mm^3^ pieces and washed in PBS. Subsequently, the samples were incubated in 800 μl papain digestion buffer [2 mM n-acetyl-l-cysteine (Sigma Aldrich), 2 mM EDTA (Carl Roth), 50 mM Na_2_HPO_4_ (Carl Roth)] and 20 μl papain solution (10 mg/ml) (Sigma Aldrich) for 24 h at 60 °C. Storage of digested tissue samples before further analysis took place at -20 °C.

DNA content was measured using the Quant-IT™ PicoGreen® dsDNA assay kit (Thermo Fisher Scientific). To achieve this, digested tissue samples (10-fold dilution for internal controls) were pipetted in 96-well plates and an equal volume of PicoGreen® reagent working solution was added. Subsequently, the samples were incubated for 5 min at room temperature, protected from light. Fluorescence (excitation wavelength of 480 nm) was measured by a microplate reader (SynergyTM H1 Hybrid Multi-Mode Microplate Reader, Gen5TM Software, BioTek® Instruments, Inc.). DNA content was determined by the use of standard curves prepared from DNA standards measured on the same plates.

Results from DNA measurement were again normalized to the respective controls and are given as percentages relative to controls.

### Assessment of cytocompatibility

#### Scaffold seeding

Allogeneic equine AT-MSC were isolated by enzymatic digestion with collagenase I (Thermo Fisher Scientific) and expanded until passage 3. Plastic-adherence, trilineage differentiation potential and expression of MSC-related surface markers were shown before further processing of the cells (data not shown). Cells cultured to approximately 60-80% of confluence were detached by trypsinization and suspended in cell culture medium for re-seeding experiments.

Scaffolds with a 2 cm^2^ surface (2 cm × 1 cm × 2 mm) from tendons that were decellularized by automated and manual protocols as described above were manually prepared using stirrup-shaped blades (Carl Roth). AT-MSC were then seeded onto the surface of the tendon scaffolds (130,000 cells in 30 μl / cm^2^ scaffold surface). After incubation at 37 °C and 5% CO_2_ for 4 h, all seeded tendon scaffolds were covered with cell culture medium and further incubated for 3 d at 37 °C and 5% CO_2_.

#### Histology and LIVE/DEAD® staining

To assess cell morphology and cell integration into the scaffold, hematoxylin and eosin staining of paraffin sections of the seeded scaffolds was performed as described above. Further, LIVE/DEAD® staining of seeded scaffolds was carried out using the staining kit [LIVE/DEAD® Viability/Cytotoxicity Kit, for mammalian cells, Thermo Fisher Scientific; calcein AM 4 mM in anhydrous DMSO, ethidium homodimer-I 2 mM in DMSO/H_2_O 1 : 4 (v/v)] according to the manufacturer’s instructions. The latter was used to evaluate cell viability and morphology as well as alignment and distribution of the seeded cells on the scaffold surface.

### Statistical analysis

Statistical data analysis was performed using SPSS® Statistics 22.0. For comparison of parameters among different groups, Friedman tests were used. Further analysis by Wilcoxon signed-rank tests was then carried out for paired comparisons. As there were no significant differences between tendon samples that underwent automated freeze-thaw cycles encased either by the plastic sampling bags or by the 15 ml centrifuge tubes, evaluation of decellularization effectiveness was performed regardless of the sample holder used. The level of significance was defined at *p* = 0.05.

## Results

### Performance of the controlled rate freezer

**Fig. 1:**
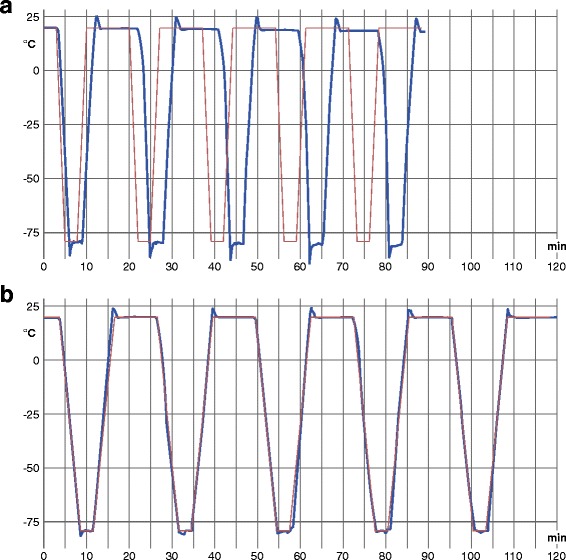
Temperature profiles of Auto-Protocol 1 (group 1) (**a**) and Auto-Protocol 2 (group 2) (**b**). Representative graphics for group 1 (**a**) (Auto-Protocol 1; cooling/heating rate of 50 °C/min) and for group 2 (**b**) (Auto-Protocol 2; cooling/heating rate of 20 °C/min). *Blue curves* represent actual values and *brown curves* show target values of the temperature. Both graphics are prepared on the basis of printed temperature records of the biological controlled rate freezer (PLANER® Kryo 360–1.7) by the use of Adobe® Illustrator® CS6 software

Graphical records printed by the controller of the controlled rate freezer plotted the actual measured temperature profile of Auto-Protocol 1 or 2 versus the programmed temperature profile (Fig. 1). The controlled rate freezer repeatably reached the required set point temperatures within the programmed period of time and with good accuracy in Auto-Protocol 2. However, in Auto-Protocol 1, which included faster cooling and heating rates, a growing delay was repeatedly observed compared to the programmed temperature profile. Furthermore, short-term temperature over- or undershoots were constantly evident at the beginning of each programmed hold at the required set point temperatures. This effect was moderate for Auto-Protocol 2 but more obvious in the records for Auto-Protocol 1.

### Effectiveness of decellularization

**Fig. 2:**
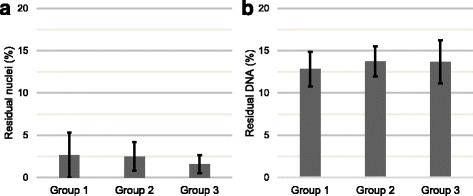
Visible nuclei count (**a**) and DNA content (**b**) of decellularized tendon samples (*n* = 10). Mean values of residual nuclei count (**a**) and residual DNA (**b**) in % relative to the controls (*n* = 10). The vertical error bars indicate the confidence interval of 95%. There were no significant differences in the number of residual nuclei and in the amount of DNA content among tendon samples of both automated protocols (group 1 and 2) and the manually performed protocol (group 3) for decellularization

All of the performed protocols resulted in both reduced cell nuclei count and reduced DNA content (*p* = 0.005) relative to respective controls, with no significant differences among samples from the three different freeze-thaw procedures (Fig. 2).

**Fig. 3:**
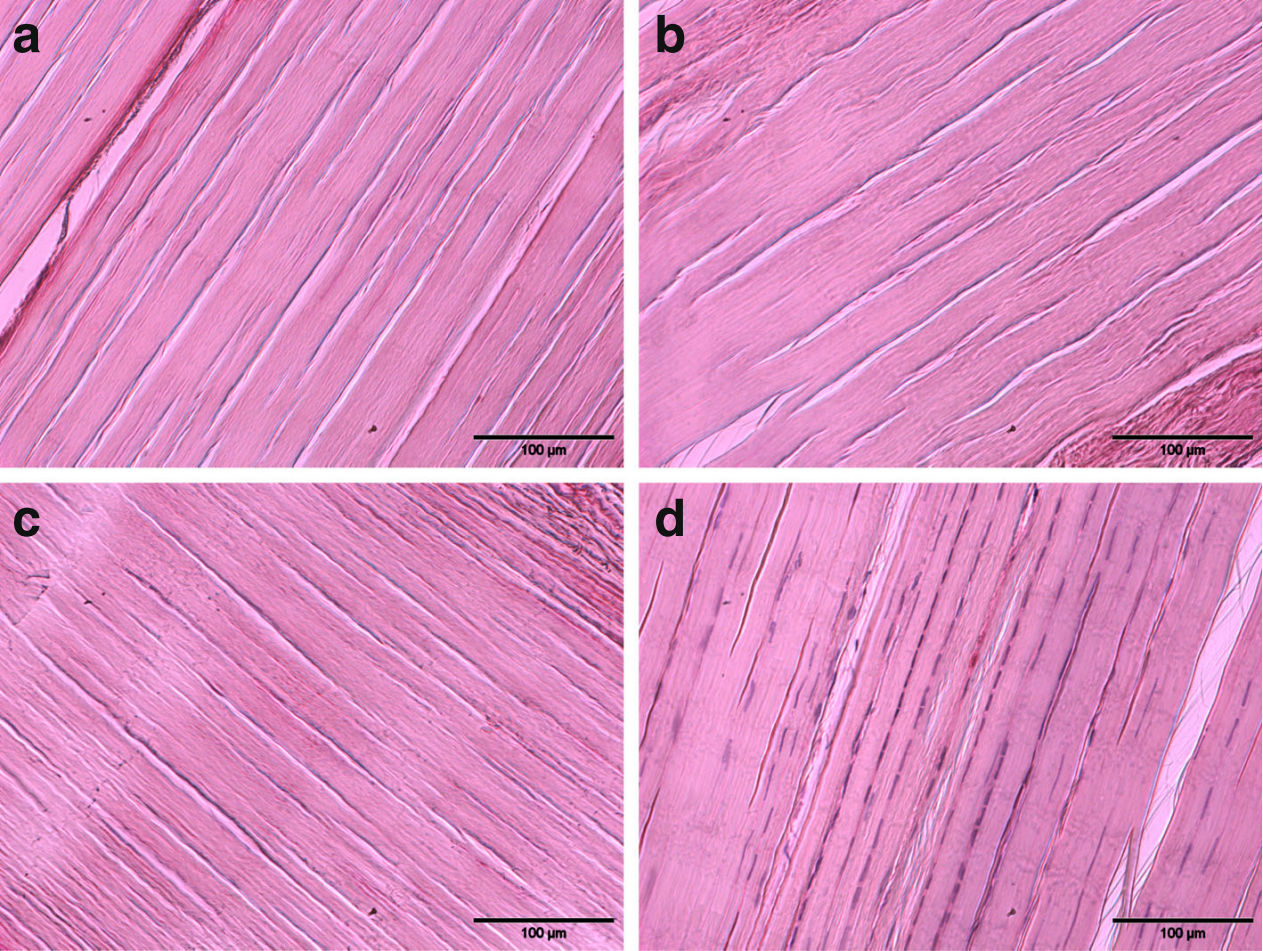
Histological assessment of decellularization effectiveness. Representative images of hematoxylin and eosin stained equine superficial digital flexor tendon samples of group 1 (Auto-Protocol 1) (**a**), group 2 (Auto-Protocol 2) (**b**), group 3 (Manual Protocol) (**c**), showing an apparent reduction of visible nuclei compared with tendon samples of the internal controls (no decellularization) (**d**). Decellularized tendon samples of all groups reveal regularly aligned collagen fibrils and interfibrillar tissue gaps instead of resident cells

Histologically, we observed very few visible nuclei within a uniformly structured ECM (Fig. 3). Empty gaps were observed, free of cellular residues, between regularly aligned collagen fibres. In contrast, histologically evaluated control scaffolds showed a preserved cellular integrity with rarely occurring signs of apoptosis. Normalized percentages of residual nuclei in tendon samples treated with Auto-Protocol 1 (mean value: 2.6%; range: 0–11.9%) and 2 (mean value: 2.5%; range: 0–6.1%) were slightly higher than those treated manually (mean value: 1.6%; range: 0–4.3%).

Mean values of residual DNA (normalized percentage values) for all applied protocols fall within a narrow range of 12.8% (Auto-Protocol 1; range: 9.2–18.2%) and 13.7% (Manual Protocol; range: 8.3–18.9%; Auto-Protocol 2; range: 9.3–16.3%). The obtained data for decellularized tendon samples correspond to a range from 9.7 to 12.7 ng DNA/mg wet weight.

### Cytocompatibility

**Fig. 4:**
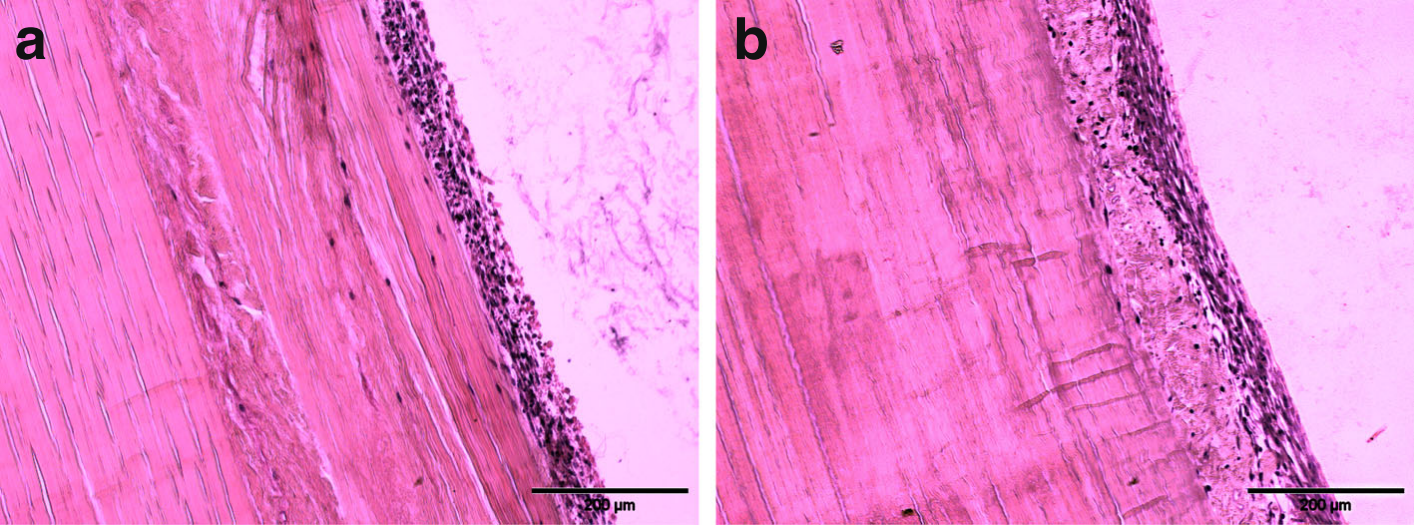
Histological assessment of cytocompatibility. Representative images of hematoxylin and eosin stained equine superficial digital flexor tendon samples after decellularization by automated (**a**) and manual (**b**) freeze-thaw cycles, re-seeding with equine adipose tissue-derived mesenchymal stromal cells and 3 days of culture. A successful re-seeding procedure of the tendon surface is indicated by the dense cell layer adhering to the sample surface, with a lower number of cells penetrating deeper tissue structures

Hematoxylin and eosin staining of re-seeded tendon scaffolds revealed successful seeding on the surface of automatically and manually processed scaffolds (Fig. 4). At day 3 after re-seeding, the majority of visible cells were attached to the scaffold surface, only a very low number of cells populated deeper tissue layers.

**Fig. 5:**
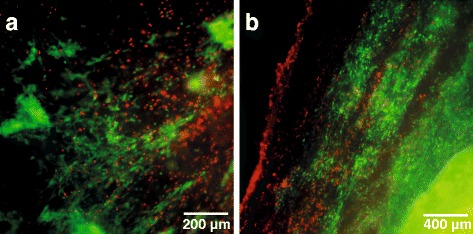
Fluorescence microscopic assessment of cytocompatibility. Representative panels of LIVE/DEAD® staining of equine superficial digital flexor tendon specimens decellularized by automated (**a**) and manual (**b**) freeze thaw cycles. Decellularized scaffolds were re-seeded with equine adipose tissue derived mesenchymal stromal cells and a fluorescence microscopic evaluation was performed after 3 days of culture. Vital cells are indicated by green fluorescence (display of intracellular esterase activity), cells with defect cellular membranes show a red fluorescence signal of their nucleus

LIVE/DEAD staining of re-seeded scaffolds showed that the majority of the cells was vital, indicated by green fluorescence, and a lower number of cells with damaged membranes indicated by red fluorescence. Morphologically, vital cells appeared elongated with collagen fibre oriented alignment, whereas roundly shaped, damaged cells showed no tendency for directed orientation (Fig. 5). The observed cell distribution was slightly inhomogeneous. Besides areas with an even distribution, there were also only sparsely populated parts of the scaffold surface.

## Discussion

The present study demonstrated the same high effectiveness of both automated and manual freeze-thaw cycles in decellularization of large tendon samples (equine superficial digital flexor tendon). Since the sole application of repeated freeze-thaw cycles for successful decellularization of natural tendon tissue has been reported as insufficient [10, 11], all protocols applied in this study were combined with a Triton X-100-based chemical treatment. Triton X-100 as a non-ionic detergent is commonly used in various decellularization protocols to solubilize cell membranes and dissociate DNA from proteins [15]. Since a lack of ionic charge results in a low impact on protein structures, non-ionic detergents are among the widely used chemical reagents for tissue decellularization [9]. However, there are conflicting data about the outcome of the decellularization effectiveness especially of Triton X-100 in current literature [16–23]. A direct comparison between treatment protocols particularly for decellularization of tendon tissue is difficult because of variations in the used concentrations, combinations and inconsistencies in data analysis and the times studied. Here, Triton X-100 was mainly chosen based on the results of our previous study, which had demonstrated the use of freeze-thaw cycles combined with this detergent to be effective for decellularization of full-thickness equine superficial digital flexor tendons and to maintain scaffold cytocompatibility [11].

Not only the generally high diversity in applied protocols for tendon decellularization, but also a lack of well-studied decellularization procedures especially for equine tendon tissue complicate a methodological comparison with focus on standardization. Referring to decellularization of equine flexor tendons, there are also successful decellularization protocols applying ionic detergents (sodium dodecyl sulfate; SDS) or organic solvents (tributyl phosphate; TnBP) as chemical agents for decellularization [23, 24]. But as far as the authors know, beside published data of Burk et al. there are only very few studies using repeated freeze-thawing for decellularization of equine flexor tendons [11, 14]. Moreover, the latter is focused on viability and biosynthesis of tendon matrix-seeded cells, rather than on a structured evaluation of decellularization effectiveness. Finally, to the best of our knowledge, there is no methodological gold standard for decellularization of full-thickness equine superficial digital flexor tendons mentioned in the literature so far.

In the present study, we inferred no significant difference between automated and manual freeze-thaw procedures with regard to histologically visible cell nuclei count. Both procedures resulted in roughly 2% remaining nuclei only. A direct comparison of histologically assessed residual nuclei to previous studies is difficult due to different scaffold size, diverse origins of the decellularized tendon and ligament tissue samples, and differing procedural steps of decellularization. The here reported results are roughly in accordance with a 100% removal of chicken flexor digitorum profundus cells by using a protocol including Triton X-100 and peracetic acid (PAA) [18]. Further, Burk et al. showed a reduction in resident cells of 99% in decellularization of equine superficial digital flexor tendon samples when processed by freeze-thawing and Triton X-100 [11]. However, protocols including Triton X-100 did not result in a successful removal of cellular remnants and led to a disrupted tendon structure in rat tail tendons and central tendon of porcine diaphragms [21, 22]. A recently published study described decellularization of equine superficial digital flexor tendon specimens in a dimension of 10 cm × 1.5 cm × 0.3 cm by using 1% TnBP in combination with 0, 1, 3 or 5% PAA. The obtained results showed an increasing reduction in histologically visible cells (63 to 99%) when treated with 0 to 5% PAA. However, the use of 5% PAA led to a significant decrease of proteoglycans and alterations of the ECM, such as opened collagen fibres as well as an increased pattern of the tendon crimp [24].

Further, the present study revealed no significant differences in DNA-content by direct comparison of automated and manual protocols, with roughly 13% residual DNA in all decellularization groups. Thereby, the presented results are in accordance with current literature [11, 18, 23]. However, Pridgen et al. showed no significant reduction in DNA content by using Triton X-100 for decellularization of human flexor tendons [16]. The latter illustrates again the high diversity in published data and the resulting difficulties in comparison of decellularization protocols for tendon tissue.

To our knowledge, this study is the first attempt to partly automate the process of decellularization of tendon tissue. Whereas currently published decellularization protocols for natural tendon tissue describe manually performed techniques for freeze-thawing steps [12–14, 23, 25–27], our technique provides a viable alternative to this strategy. Due to the more standardized and optimized processing with continuous documentation, automation of repetitive freeze-thawing procedures appears as a vital step for the development of future decellularization protocols.

Usually, controlled rate freezers are utilized in the field of cryopreservation [28] and for experimental studies that evaluate the effects of freezing on diverse tissue and cell characteristics [29], focusing on preserving vitality in terms of storage. Therefore, these machines are designed for relatively slow cooling procedures, as required for gentle freezing of cells. This is in accordance with the here reported temperature profiles of the controlled rate freezer that showed a more precise course of temperature for slower cooling rates.

On the contrary, the present study utilized a conventional liquid nitrogen-based controlled rate freezer for physical decellularization of natural tendon tissue, for which rapid freeze-thawing is commonly used. The desired effect of freezing in terms of decellularization is a direct cell injury with minimal alteration of ECM composition. Since all steps of freeze-thawing, including cooling rate as well as the coldest set point temperature, freezing hold time, thawing rate and number of repetitions, are able to influence cell injury [30], the present study applied two different cooling and heating rates. Their direct comparison revealed no significant difference in their effectiveness for decellularization of tendon samples. A slow freezing rate, associated with extracellular ice crystal formation and a subsequent hyperosmolar shift in the extracellular environment, leads to cell dehydration as well as intracellular solute concentration, which is not always lethal for resident cells. Conversely, faster cooling rates are associated with intracellular ice crystal formation, leading almost always to cell death due to disintegrated organelles and cell membranes [30]. However, the obtained data suggest a minor importance of the applied cooling rate for decellularization effectiveness in large tendon samples. For further interpretation and especially for a comparison to freeze-thawing-associated effects in other connective tissue types, it should be noted that response to and threshold for cooling rates are cell-specific features [31, 32]. Moreover, the disrupting effects of intracellular ice crystals depend on a sufficient duration of thawing [30]. Finally, in this study, the controlled rate freezer accurately performed slow cooling and heating rates of -20 °C per min and +20 °C per min. As this procedure was effective for decellularization of large tendon samples, the utilized controlled rate freezer is suitable for this purpose. A possible methodological refinement is related to the requirement of liquid nitrogen. Given the problematic risk of contamination during transport or storage, the use of liquid nitrogen under GMP conditions is limited [28]. Therefore, future investigations aiming at a more extensive sample processing may benefit from alternative strategies providing cryogenic temperatures.

For further characterization of the decellularized tendon scaffolds, the present study provided a basic assessment of cytocompatibility by re-seeding the tendon surface with equine AT-MSC. In the field of tissue engineering, re-seeding procedures of decellularized tendon matrices are still challenging since their dense structure makes a satisfactory cell infiltration into deeper tissue layers complicated [8]. Nevertheless, promising results of cell seeded scaffolds for tendon regeneration justify the search for more appropriate re-seeding techniques [33–36]. In the present study, tendon samples that underwent automated as well as manual freeze-thaw cycles could successfully be re-seeded. However, as reported before, only a very low number of cells penetrated deeper tissue layers, emphasizing the need for improved re-seeding techniques. The latter should aim for residual-free tendon scaffolds with a loosened matrix to allow ingrowth of scaffold-seeded cells into an almost completely preserved tissue-specific ECM. Among already published technical approaches promising effects resulted especially from using ultrasound sonication, PAA treatment and several injection techniques [1, 8, 37–39]. Further, the reduction in scaffold dimension to a thickness of 300 μm led to an improved tissue penetration of cells in the present experimental set-up (unpublished data) as well as in already published studies [25]. Although the majority of cells seeded on the tendon scaffolds was vital, there were also roundly shaped, damaged cells observed by LIVE/DEAD staining in the present study. Referring to possible causes that led to damaged and detached cells, future re-seeding procedures should reduce manual handling of the seeded constructs. Other factors that mainly influence cell viability include chemical residues with cytotoxic effects and the initial cell seeding density. Particularly, metabolic stressors such as nutrient availability and metabolic byproducts of cellular origin gain in importance as cell density increases [40].

The horse is referred to as the fourth most frequently used source of tendon tissue for decellularization [5]. In terms of translation, the equine superficial digital flexor tendon is considered to be particularly important since its functional, structural, and pathological characteristics have already been subject of extensive research [41, 42] and are similar to those of the human Achilles tendon. Therefore, the horse is considered as the ideal animal model for human tendon pathologies, such as exercise-induced Achilles tendon injury [43]. With regard to providing commercially available tissue grafts for xenotransplantation, equine tendon tissue offers further benefits. Those include the accessibility related to breeding and slaughter, the provided dimension of tendon tissue required in reconstructive surgery, and the limited number of zoonotic agents [5].

Future studies should aim to assess further characteristics of tendon matrices that underwent automated freeze-thaw cycles. These could include an evaluation of ECM ultrastructure and biochemical composition, of mechanical properties, of antigen removal and of adverse host reactions induced by the tendon scaffolds. To allow the production of commercially available tendon graft materials for use in human as well as in veterinary medicine, high quality manufacturing processes are required. Therefore, automation of so far manually performed key procedures in tissue decellularization, such as freeze-thawing, is essential. Moreover, future methodological research should focus on the automation of further decellularization steps. The latter especially include automated washing steps under continuous agitation performed in a closed system and applicable for a high number of samples. Finally, increased requirements with regard to validation, reproducibility, and safety, need to match with practicable approaches.

## Conclusion

Automated freeze-thaw cycles carried out by a liquid nitrogen-based controlled rate freezer are effective for decellularization of large tendons when combined with a non-ionic detergent treatment (Triton X-100). These findings are an essential step towards a standardized production of decellularized tendon scaffolds for various in vitro applications and further development of graft-based reconstruction of musculoskeletal defects.

## Abbreviations

AT - MSC: Adipose tissue-derived mesenchymal stromal cells
ECM: Extracellular matrix
FBS: Fetal bovine serum
GMP: Good manufacturing practice
PAA: Peracetic acid
PBS: Phosphate buffered saline
SDS: Sodium dodecyl sulfate
TnBP: Tributyl phosphate
